# Supplementary material for: Upregulation of LAMB1 via ERK/c-Jun Axis Promotes Gastric Cancer Growth and Motility
Source: Int J Mol Sci. 2021 Jan 10;22(2):626. doi: 10.3390/ijms22020626 (PMC7826975; doi:10.3390/ijms22020626)
Supplement: Supplementary file 1 [file ijms-22-00626-s001.pdf]

Supplementary Information

Supplementary tables

Table S1. Enriched KEGG pathway identified in upregulated DEGs

KEGG pathway	Gene count	P-value	Gene symbol
ECM-receptor interaction	11	6.30E-12	COL1A1, COL1A2, COL3A1, COL4A1, COL4A2, COL5A2, COL6A3, ITGB1, **LAMB1**, SSP1, THBS1
Focal adhension	12	2.10E-09	COL1A1, COL1A2, COL3A1, COL4A1, COL4A2, COL5A2, COL6A3, CCND2, ITGB1, **LAMB1**, SPP1, THBS1
PI3K-Akt signaling pathway	14	3.60E-09	COL1A1, COL1A2, COL3A1, COL4A1, COL4A2, COL5A2, COL6A3, CCND2, CCNE1, HSP90AB1, ITGB1, **LAMB1**, SPP1, THBS1
Protein digestion and absorption	8	1.80E-07	ATP1B3, COL1A1, COL1A2, COL3A1, COL4A1, COL4A2, COL5A2, COL6A3
Amoebiasis	7	1.10E-05	COL1A1, COL1A2, COL3A1, COL4A1, COL4A2, COL5A2, **LAMB1**
Small cell lung cancer	5	7.60E-04	COL4A1, COL4A2, CCNE1, ITGB1, **LAMB1**
Pathways in cancer	8	2.60E-03	COL4A1, COL4A2, CCNE1, HSP90AB1, ITGB1, **LAMB1**, MMP1, STAT1
Toll-like receptor signaling pathway	3	9.60E-02	CTSK, SPP1, STAT1

Table S2. Enriched Gene Ontology identified in upregulated DEGs.

Gene Ontology	Gene count	P-value	Gene symbol
**BP (biological process)**			
Extracellular matrix organization	15	1.5E-15	COL1A1, COLA2, COL3A1, COL4A1, COL4A2, COL5A2, COL6A3, ITGB1, **LAMB1**, LUM, NID2, SPP1, SPARC, SERPINB5, THBS1
Collagen catabolic process	10	5.2E-13	CTSK, COL1A1, COL1A2, COL3A1, COL4A1, COL4A2, COL5A2, COL6A3, MMP1, MMP7
Cellular response to amino acid stimulus	5	1.7E-05	COL1A1, COL1A2, COL3A1, COL4A1, COL5A2
Extracellular matrix disassembly	5	1.1E-04	BMP1, CTSK, MMP1, MMP7, SPP1

Cell-matrix adhesion	3	3.5E-02	COL3A1, ITGB1, NID2
Endothelial cell migration	2	9.1E-02	FAP, STAT1
**MF (molecular function)**			
Extracellular matrix structural constituent	8	1.5E-09	COL1A1, COL1A2, COL3A1, COL4A1, COL4A2, COL5A2, **LAMB1**, LUM
Laminin binding	3	2.8E-03	LGALS1, ITGB1, THBS1
Extracellular matrix binding	3	3.0E-03	CTSK, ITGB1, THBS1

Table S3. (related figure 1D) List of the relative expression genes from GSE datasets.

Number	Gene symbol			
	**GSE2685**	**GSE13861**	**GSE33651**	**GSE63089**
1	MEST	CLDN1	CBWD3	PBK
2	HSPA1B	COL1A2	COL1A2	DEPDC1B
3	HOXB7	BGN	DNAJA1	TNS4
4	MIPEP	COL4A1	COL4A2	FN1
5	GGH	RNF24	PTPN6	CDCA7
6	TFRC	GRINA	**LAMB1**	MPZL1
7	IFT88	CDK1	TNFSF13B	**LAMB1**
8	SUZ12	ACTC1	ATP5A1	PRDX1
9	COL5A2	CDK4	NDUFV2	USP7
10	**LAMB1**	**LAMB1**	PGK1	DYNLL1
11	USP14	NUP35	PSMF1	CISD2
12	TSN	NCAPG2	ANKRD28	TRAIP
13	KCNA5	HAVCR2	RPS27	ABCC4
14	HYOU1	HIF1A	KLF6	TMA16
15	CDK16	CECR5	CD2	QSER1
16	CDC25C	SHC1	SEC24C	LAMB3
17	HIST2H4	CYTH2	HERC5	WBP1L
18	PPP2R2A	ZNF670	TSPAN14	RUNX3
19	CCT2	TBKBP1	PRR14	AMELX
20	PTDSS1	TRIM27	PDS5B	KLC4

Supplementary figures

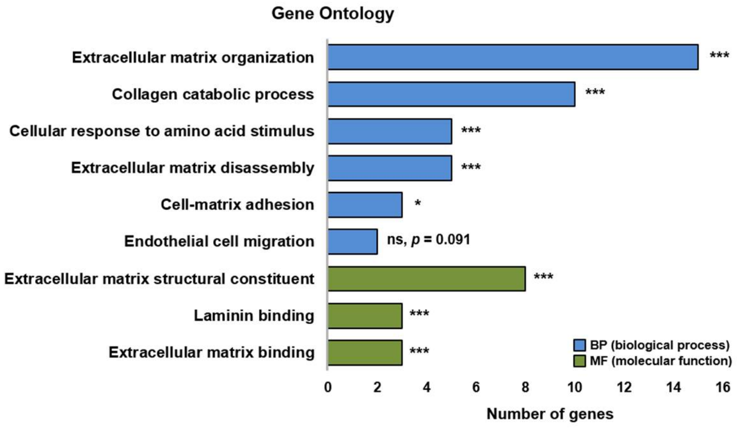

Figure S1. Gene ontology (GO) enrichment analysis of upregulated genes using public database of GSE data. The data is arranged in order of low p-value. $^{*}\ p < 0.05$; $^{***}\ p < 0.001$, ns: not significant.

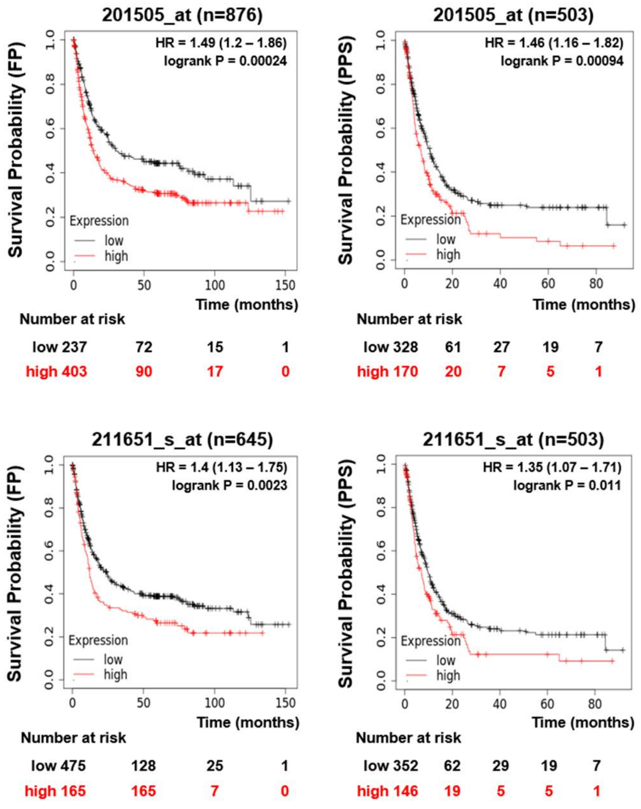

Figure S2. LAMB1 is related to poor prognosis in gastric cancer patients. Kaplan-Meier plots of the first progression (FP) and post progression survival (PPS) for LAMB1 expression in public database of gastric cancer patients are presented.

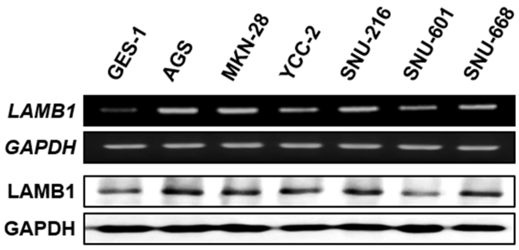

Figure S3. LAMB1 is upregulated in gastric cell lines. The mRNA and protein expression levels of LAMB1 in gastric epithelial cell and six gastric cancer cell lines are presented.

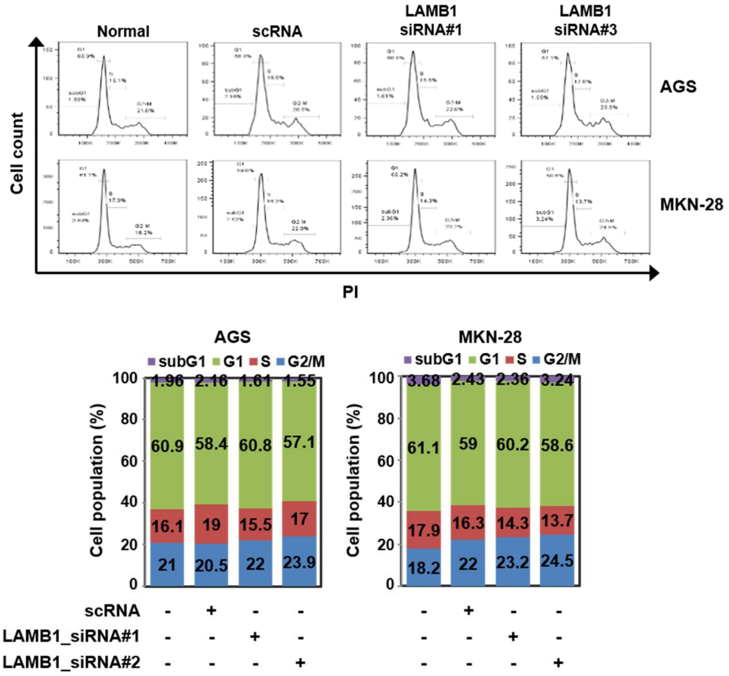

Figure S4. Silenced LAMB1 does not induce cell cycle arrest of AGS and MKN-28. Cell cycle analysis of AGS and MKN-28 cells transfected with scRNA or siRNA for LAMB1 knockdown was performed. Percentage of cell population is presented in the bottom panel.

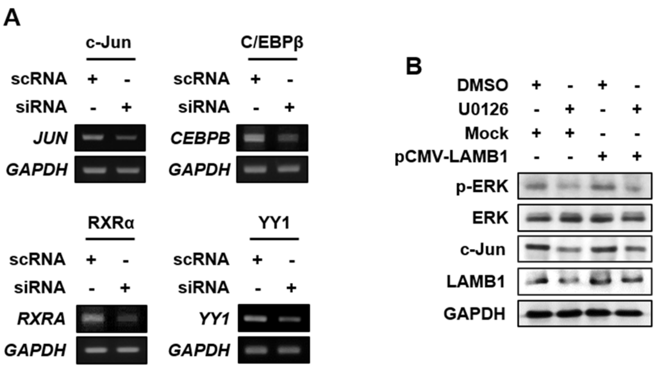

Figure S5. c-Jun knockdown suppresses biological function of AGS through LAMB1. (A) The mRNA expression in AGS cells using scrambled siRNA (scRNA) or various siRNA transfection. (B) The expression of c-Jun in AGS cells transfected with pCMV-3Tag-1A vector (Mock, empty vector) or pCMV-3Tag-1A-LAMB1 (pCMV-LAMB1, LAMB1 overexpression vector) after pretreatment with 10 μM U0126 for 24 h.
